# Gastrointestinal Symptoms in Autism Spectrum Disorder: A Systematic Review

**DOI:** 10.3390/nu14071471

**Published:** 2022-04-01

**Authors:** Geraldine Leader, Cathal Abberton, Stephen Cunningham, Katie Gilmartin, Margo Grudzien, Emily Higgins, Lokesh Joshi, Sally Whelan, Arlene Mannion

**Affiliations:** 1 Irish Centre for Autism and Neurodevelopmental Research, School of Psychology, National University of Ireland Galway, University Road, H91 TK33 Galway, Ireland; cathalabb@gmail.com (C.A.); stephen.cunningham@nuigalway.ie (S.C.); katiegilmartin1@hotmail.com (K.G.); emily.higgins@nuigalway.ie (E.H.); lokesh.joshi@nuigalway.ie (L.J.); sally.whelan@nuigalway.ie (S.W.); arlene.mannion@nuigalway.ie (A.M.); 2 Healthy Mind Clinic, 61 Old Church Crescent, Clondalkin, D22 VK63 Dublin, Ireland; mindfultherapistdublin@gmail.com

**Keywords:** autism spectrum disorders, gastrointestinal symptoms, comorbidity, regression, language and communication, severity, challenging behavior, psychopathology, sleep problems, sensory issues, autism symptoms

## Abstract

This systematic review aims to offer an updated understanding of the relationship between gastrointestinal symptoms (GIS) and autism spectrum disorder (ASD) in children and adolescents. The databases PsycINFO, Medline, Cinahl, and ERIC were searched using keywords, and relevant literature was hand-searched. Papers (*n* = 3319) were systematically screened and deemed eligible if they were empirical studies published in English since 2014 and measured the GIS of individuals with ASD who were under 18 years old. Thirty studies were included in the final review. The study findings were synthesized under eight themes, including the prevalence and nature of GIS and their relationship with developmental regression, language and communication, ASD severity, challenging behavior, comorbid psychopathology, sleep problems, and sensory issues. The review found that GIS were common and that there was contradictory evidence concerning their relationship with co-occurring conditions. It also identified evidence of some causal relationships that support the existence of the gut–immune–brain pathways. Future research needs to use large prospective designs and objective and standardized GIS measurements to provide a nuanced understanding of GIS in the context of ASD.

## 1. Introduction

### 1.1. Gastrointestinal Symptoms in Autism Spectrum Disorder

Autism spectrum disorder (ASD) is a neurodevelopmental disorder that presents with restrictive, repetitive patterns of behavior, interests, and activities, and/or deficits in communication and social interactions, which typically manifest within the first three years of life [1]. Individuals with ASD frequently have comorbidities [2] and they are at greater risk of experiencing co-occurring gastrointestinal (GI) symptoms, including constipation, diarrhea, and abdominal pain [3]. GIS are experienced by between 9 and 91% of children with ASD, and individuals with GIS have a lower quality of life compared to that of those with no GIS [4]. GIS can cause pain and distress and individuals who have little to no communication skills and may not be able to tell their caregivers that they are in pain [5]. Abdominal pain may also act as a trigger for challenging behavior [6]. Challenging behaviors are more frequent in children with ASD who also experience abdominal pain, diarrhea, and constipation. Additionally, individuals with ASD with GIS can be more irritable, withdrawn, or hyperactive compared to those without GIS [7,8]. Abdominal pain and constipation have also been found to predict challenging behavior, the presence of diarrhea predicts tantrum behavior, and nausea predicts worrying/depression and avoidant behavior [9]. In addition, children with sleep abnormalities are more likely to have GI problems than those who have good sleep quality [10]. Chronic GI problems have also been associated with higher levels of sensory over-responsivity [11].

There is growing evidence that GI disorders can arise due to genetic and environmental risk factors for ASD [12,13]. However, the etiology of GIS in ASD remains poorly understood [14]. A detailed understanding of the nature of GIS and how they are associated with co-occurring conditions is required to facilitate the diagnosis of ASD and the treatment of GIS [15].

A literature review by Mannion and Leader examined twenty-eight studies that focused on the relationship between GIS and developmental regression, language, ASD severity, challenging behavior, comorbid psychopathology, sleep problems, and sensory issues [4]. The term regression, in relation to ASD, refers to the loss of previously used or developed skills such as motor, social, and/or language skills [16]. Mannion and Leader [4] identified in 2014 that contradictory evidence existed about the prevalence of GIS in relation to language regression, communication, and ASD severity. For example, children with ASD who present with language regression experience more GIS than those without language regression [17] and yet, a history of regression is not significantly associated with current and past GIS [18,19]. In relation to communication, Gorrindo et al. [20] found that younger, more socially impaired, and nonverbal children have an increased likelihood of constipation. In contrast, Chandler et al. [18] and Williams et al. [21] found no difference between the verbal ability of children with and without GIS. Similar contradictions are evident regarding whether the presence and frequency of GIS are associated with ASD symptom severity. Some studies found that GIS are related to the severity of ASD [22], whereas other research found current or previous GIS have no significant association with ASD severity [18]. To address these contradictions, Mannion and Leader [4] argued that more rigorously designed studies were needed to identify the risk factors of GI disorders, atypical presentations of GI disorders in ASD, and subpopulations within ASD that experience GIS.

### 1.2. Current Study

To address contradictory evidence and the resulting gap in knowledge discussed above, the current systematic review aimed to update the review of Mannion and Leader [4], to provide a state-of-the-art understanding of the frequency and nature of GIS in ASD, and to identify the key factors that are associated with their presentation. Given the recent emphasis on the gut–immune–brain axis pathways in the development of ASD in young children, the current review, in contrast to that of Mannion and Leader [4], focuses solely on research conducted on children and adolescents.

## 2. Materials and Methods

### 2.1. Search Procedures

A systematic search was conducted to identify literature in the following four databases: PsycINFO, Medline, Cinahl, and ERIC. Each database was searched independently by three researchers (EH, KG, and CA), using combinations of the following key terms: gastrointestinal, constipation, abdominal pain, abdominal bloating, diarrhea, nausea, vomiting, encopresis, gastroesophageal reflux, gaseousness, food regurgitation, quality of life, sleep problems, sensory issues, challenging behavior, communication, developmental regression, ASD severity, comorbid psychopathology, ASD, and developmental disability. Searches were limited to peer-reviewed papers written in English. Because Mannion and Leader [4] had previously reviewed papers published in 2013, the current review limited searches to papers published between the years 2014 and 2021. From the databases, 3308 were papers initially identified. Hand-searching the reference lists of relevant papers identified eleven additional papers. After the removal of duplicates, 2726 papers remained.

### 2.2. Eligibility Criteria and Paper Selection Process

**Figure 1:**
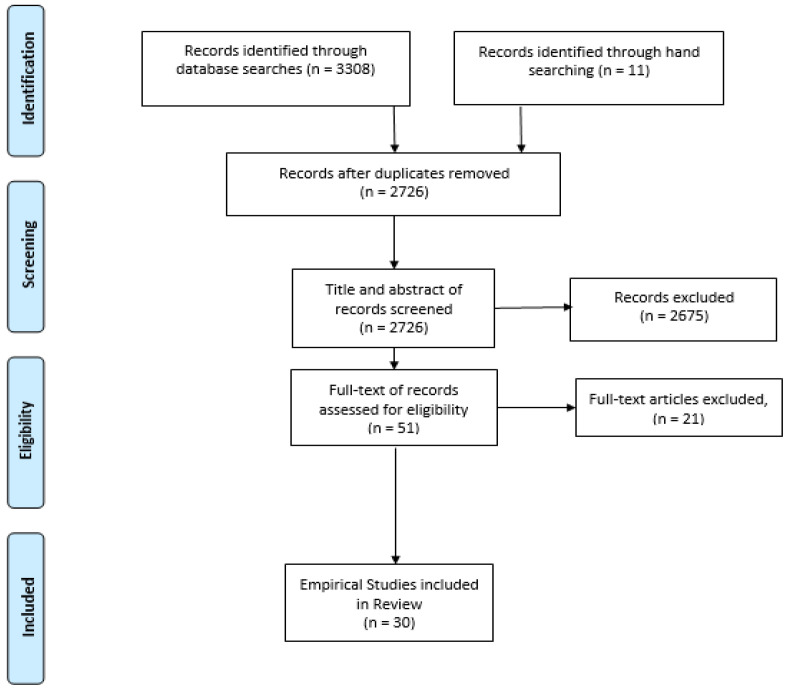
Flow diagram of paper selection.

Researchers (EH and KG) initially examined the items independently. They screened the items for eligibility according to predetermined eligibility criteria. Items were included if they were empirical studies that involved children and adolescents under 18 years who were diagnosed with ASD and if they focused on the measurement of GIS. Papers were excluded if they involved people over 18 years old; they were published before 2014; they focused on the psychometric evaluation of measurement tools; and if they were review papers, case reports, editorials, letters, commentaries, or reviews. After screening the titles and abstracts of the items, 593 were retained. Then, the full texts of these items were obtained and screened against the eligibility criteria. Having worked independently, EH and KG worked together and calculated their inter-rater agreement on 25% (*n* = 681) of the search results. This revealed an 87% agreement, which was fair (Cohen’s κ = 0.40). The researchers discussed their decisions concerning all the items upon which they had disagreed regarding eligibility. If eligibility was not agreed upon through this discussion, other researchers (CA and SW) made the final decisions about whether these papers met the eligibility criteria. The search results and the paper’s selection processes are summarized in a PRISMA flow diagram presented in Figure 1.

### 2.3. Quality Appraisal and Data Extraction

Data were extracted from the included studies to identify and record the study aims, sample size, age of participants, the tools used to measure GIS, and the study results. The strengths and weaknesses of the studies were appraised using the Quality Assessment for Diverse Studies (QuADS) tool [23]. This tool has demonstrated good inter-rater reliability and face and content validity [23]. Prior to conducting the quality appraisals, researchers (EM, KG, and CA) examined and discussed the QuADs criteria. Then, the researchers independently applied the QuADs criteria to three studies. Following this, they met again to discuss their scoring and to identify any inconsistencies in their interpretation of the QuADS criteria. Inter-rater reliability identified a 74.36% agreement rate. Following discussion, the researchers achieved 100% consensus on how to apply the QuADS tool. Then, independently, they applied the criteria to all the remaining studies. Afterwards, the researchers met again and discussed their quality appraisal ratings on all the studies until they achieved 100% agreement.

## 3. Results

### 3.1. Summary of Studies

Thirty papers were included in the final review. Table 1 provides a summary of the study details and the QuADS results. The studies were reviewed, and their findings were synthesized into seven themes that are reported below: The Prevalence and the Nature of GIS; GIS and ASD Severity; GIS and Regression; GIS and Language/Communication; GIS and Challenging Behavior; GIS and Comorbid Psychopathology; GIS and Sleep Problems; GIS and Sensory Issues.

### 3.2. The Prevalence and the Nature of GIS

Studies vary regarding the proportion of children with ASD in samples reported to experience at least one GIS. The prevalence of GIS in autistic samples has been reported to be 30–37.4% [30,59] and 82.4% in the previous three months [41]. Whereas Khalil et al. [40] reported that all their autistic sample (*n* = 58) had at least one GIS. In contrast, a large epidemiological study involving *n* = 1244 children with ASD found GIS were reported for 43.1% of children with ASD, aged 2–5 years.

The studies in this review found that children with ASD are significantly more likely to experience GIS than control groups and children with typical development (TD) [8,24,26]. Chaidex et al. [8] found children with ASD were at least three times more likely to experience a higher frequency of most GIS than children with TD. This finding was supported by a large prospective study that examined GIS frequency in ASD (*n* = 195) in comparison to TD (*n* = 4095) and children with other developmental disabilities (DD) (*n* = 4636) [26]. In this study, mothers were twice as likely to report GIS in children with ASD than in those with DD and TD. Children with ASD aged 6–18 months were more likely than those with TD to report constipation (adjusted odds ratio (aOR), 2.7; 95%CI, 1.9–3.8; *p* < 0.001) and food allergy/intolerance (aOR, 1.7; 95%CI, 1.1–2.6; *p* = 0.01). Parents of children with ASD aged 18–36 months were more likely to report diarrhea (aOR, 2.3; 95%CI, 1.5–3.6; *p* < 0.001), constipation (aOR, 1.6; 95%CI, 1.2–2.3; *p* < 0.01), and food allergy/intolerance (aOR, 2.0; 95%CI, 1.3–3.1; *p* < 0.01).

There is contradictory evidence regarding whether the prevalence of GIS differs according to gender. Several studies have reported no significant differences in gender, for children with and without GIS [48,49,50]. However, other studies have reported gender differences in the frequency of GIS. Babinska et al. [24] found GIS more common in girls with ASD than in boys (97.1% to 87.6% 2 = 13.57, *p* = 0.009), and 70.6% of girls and 44.5% of boys experienced GIS several times weekly or every day. Mazefsky et al. [44] also found that a significantly higher proportion of females than males had GIS, *p* < 0.05.

The evidence suggests that the prevalence of symptoms is not associated with the age of children with ASD. No difference in the prevalence of GIS was noted after adjusting for age [8,14], and younger and older children had similar rates of four common types of GIS (p’s > 0.05) [14]. Furthermore, GIS persist over time for individuals as they increase in age [24]. Bresnahan et al. [26] found GIS that existed between the ages of 6 to 18 months were also present between 18 and 36 months. Change in GIS over time has also been examined to assess whether comorbid symptoms persist or change and to determine if a relationship exists between family medical history, history of autoimmune diseases, and child comorbid conditions [43]. This follow-up study was conducted two years after the initial research took place in 2013. The original study had involved n = 89 children and adolescents, and the follow-up included n = 56 of the original children. Results of the follow-up indicated that GIS continued in 84.4% of participants. However, there was a significant difference between overeating at baseline and at two-year follow-up when overeating had become more severe. The majority of participants (92.9%) presented with a family history of autoimmune disease which was most commonly osteoarthritis, psoriasis, and hypothyroidism. They also reported a relationship between thyroid disorders and GIS.

The type of GIS experienced by children with ASD can be divided into those that affect the upper and lower GI tract, and more generalized GI discomfort/pain. The evidence regarding the prevalence of GIS affecting the upper GI tract reveals that reflux was found in 5.5% of participants [46], but in half of a sample of children with ASD (*n* = 18) who had experienced reflux, this symptom had resolved in children aged 25–98 months [8]. Nausea was reported in 23.2% [14] and 27.9% [41] of the samples. Whereas vomiting was reported in between 4.2% and 11.4% of the samples [46,50]. Symptoms affecting the lower GI tract include constipation, diarrhea, and pain on stooling. Constipation occurred in 22.1–24.1% of children with ASD in some samples [46,48] and 47.1–65% in other samples [14,24,41,50]. Pain on stooling affected between 7.4% and 29.5% of two samples of children with ASD [48,50]. The prevalence of diarrhea symptoms ranged between 10.6% and 29.7% [14,40,46] and 40.4–64.7% [41,50]. Khalil et al. [40] also noted that 86.2% of their sample experienced offensive stools. Symptoms of discomfort and pain throughout the GI tract were described as stomachache and/or abdominal pain and bloating. These symptoms were reported to affect between 22.9% and53.7% [14,41,50] and 24.3–44.3% [46,50], respectively. Chaidex et al. [8] investigated how GIS are combined in individuals. They reported that when individuals experienced one or more symptoms, diarrhea and constipation did not tend to be experienced in the same individuals. Only nine children with ASD were reported to have both diarrhea and constipation in the previous three months. In contrast, constipation co-occurred with other GIS in the same children, and 29 children had diarrhea in combination with other GIS.

Several studies have reported the number of GIS types experienced by individual children with ASD. Ferguson et al. [14] found that the number of GIS ranged from one to four, with an average number of 1.66 (SD = 0.880). In other studies, the number of individuals with two symptoms ranged from 8.3T to 22.1% [41,46]; three symptoms 3–22.1% [41,46]; four or more symptoms 2.6–22% [41,46]. GIS were experienced several times a week or daily by 47.6% of children with ASD [24]. Neuhaus et al. [46] found severe abdominal pain in 5.1% of their autistic sample, whereas Prosperi et al. [48] reported that 25.8% experienced at least one severe GIS.

### 3.3. GIS and ASD Severity

Several studies have found that ASD severity is unrelated to GIS [36,40,44,50]. Khalil et al. [40] found a positive but not significant relationship between GIS and ASD severity. Jiang et al. [36] also found no association between small samples of age and gender-matched groups of children with ASD aged 17–37 months, with (*n* = 28) and without GIS (*n* = 28). However, Yang et al. [59] found that children with and without GIS had significantly different levels of social interaction and stereotyped behaviors.

Further evidence of the relationship between GIS and ASD symptoms and the existence of a gut–immune–brain axis was provided by studies that have investigated differences in the gut microbiota composition of children with ASD and the presence of GIS. Rose et al. [52] investigated differences in biological signatures in terms of immune dysfunction and microbiota composition in a sample of children with ASD (*n* = 102) aged from 3 to 12 years old. The results showed the microbiota composition of the children with GIS differed from that of those without GIS and they produced increased levels of mucosa-relevant cytokines. Rose et al. concluded that this suggested that chronic gut inflammation plays a part in ASD pathogenesis. Tomova et al. [56] also provided evidence that the gut microbiota plays a role in ASD. They identified changes in the fecal microbiota in children with ASD to determine its role in the development of GI disorders and other manifestations of ASD. The fecal microflora of a small sample of children with ASD (*n* = 10), siblings (*n* = 9), and healthy children (*n* = 10) was investigated, and parental questionnaires were used to collect data on GIS. The fecal microbiota of the children with ASD showed a significant decrease of the Bacteroidetes/Firmicutes ratio, and an elevated amount of Lactobacillus spp. Results also showed a trend in the incidence of elevated *Desulfovibrio* spp. in children with ASD. In addition, there was a very strong association of the amount of *Desulfovibrio* spp. with the severity of ASD according to the Autism Diagnostic Interview (ADI) restricted/repetitive behavior subscale score [62]. The participants demonstrated a strong positive correlation of ASD severity with the severity of GI dysfunction. Supplementing the diet with probiotics normalized the Bacteroidetes/Firmicutes ratio, *Desulfovibrio* spp., and the amount of Bifidobacterium spp. in feces of children with ASD. Shaahan et al. [54] also evaluated the efficacy and tolerability of a probiotic in children with ASD (*n* = 30; 5–9 years; mean age (84.77 ± 16.37 months)). In this small trial with no placebo group, assessors were unblinded, and participants were receiving behavioral therapy. Children with ASD were gender/age-matched with healthy controls who were relatives. Three probiotic strains of Lactobacillus acidophilus were administered for 12 weeks. After the treatment period, there were significant improvements in GIS with reductions in constipation (42.5%), diarrhea (37.5%), abdominal pain (60%), flatulence (57%), and improved stool consistency (16.6%). There were also significant improvements in ASD severity concerning speech and language communication, sociability, sensory cognitive awareness, health physical behavior.

Long-term improvements on GIS and ASD symptoms have also been found following interventions designed to normalize gut microbiota using microbiota transfer therapy (MTT). Kang et al. [37] investigated the safety and tolerability of MTT and its effects on microbiota, GIS, and other ASD-related symptoms. The study involved participants with ASD (*n* = 18) aged 7 to 17 years. Treatment involved a two-week antibiotic, a bowel cleanse, and then an extended fecal microbiota transplant (FMT). The results indicated an 80% reduction of GIS at the end of treatment, with improvements in symptoms of constipation, diarrhea, indigestion, and abdominal pain. These improvements persisted for eight weeks after treatment. Clinical assessments demonstrated that the behavioral symptoms of ASD improved significantly and that these remained improved at eight weeks follow-up. Kang et al. [38] report that these improvements were maintained two years after the treatment phase was completed. Grimaldi et al. [34] conducted a small double-blind placebo RCT that examined multiple interventions. Participants were randomly assigned for six weeks to a prebiotic and exclusion diet (*n* = 12) or a placebo and exclusion diet, or a prebiotic and unrestricted diet (*n* = 18). Those taking the prebiotic and exclusion diet had significantly lower abdominal pain (*p* < 0.05), bowel movements (*p* < 0.001), a significant increase of Lachnospiraceae family, significant changes in fecal and urine metabolites, and reduced antisocial behavior. Furthermore, the exclusion diet and prebiotic group had a higher abundance of B. Longum. Grimaldi et al. suggested that a synergistic effect on behavior resulted from the combination of prebiotic and casein/gluten-free exclusion diet.

There is some evidence that suggests the relationship between GIS and ASD severity may be impacted by the presence or absence of developmental delay, and which GIS is being examined. Chaidex et al. [8] compared children with ASD who were with and without developmental delay. They found that GIS did not significantly differ between groups regarding total GIS. However, there was a higher occurrence of vomiting in children with ASD without developmental delay, whereas vomiting was infrequently reported in children with ASD with developmental delay. In contrast, children with more severe ASD symptoms had 10% more frequent diarrhea (16.1% vs. 6.4% *p* = 0.002) than those with fewer ASD symptoms.

### 3.4. GIS and Regression

Reynolds et al. [51] recently examined the association between GIS and developmental regression in a multisite community-based case-control study that involved preschool children with ASD (*n* = 672). Developmental regression was determined using the ADI-R [63]. They found that children with developmental regression had more GIS (42.9%) than those without (31.8%), adjusted OR (95% CI) 1.53 (1.33–1.77). In particular, children with regression were more likely to have diarrhea/loose stools and vomiting. However, the same children had slightly decreased odds of having constipation.

### 3.5. GIS and Language/Communication

Two studies have investigated GIS and their association with language and communication ability. Neumeyer et al. [47] conducted a large study with children registered with the 15 Autism Speaks, autism treatment network registry sites. They used clinician-reported diagnoses and examined whether co-occurring medical conditions commonly coexist in the same children, in two ages groups of children under (*n* = 2114) and over six years old (*n* = 1221). This study found that constipation was associated with speech problems in both age groups: (younger children, 8.09%; O/E = 1.20; *p* < 0.001; older children, 6.39%; O/E = 1.21; *p* < 0.001). In addition, speech problems were also associated with feeding problems: (younger children, 9.74%; O/E = 1.23; *p* < 0.001; older children, 4.42%; O/E = 1.27). These findings are in contrast to those of Prosperi et al. [48] who found no difference in the verbal ability of children with and without GIS. Prosperi et al. [49] used parental reports to examine associations between verbal and nonverbal children with ASD with GIS (*n* = 30) in comparison to those without GIS (*n* = 55). They found that associated behaviors included frequent clearing of throat, swallowing and/tics, signing and/or whining; moaning and/or groaning, and aggressive behaviors. Children differed in the modality that they used to express discomfort according to their verbal ability. Those without verbal skills used screaming, sighing, and/or whining and moaning and/or groaning more to express their discomfort. They concluded that associated behaviors may be useful to identify GIS in nonverbal children and that they may help develop objective measures to identify GIS in those who are nonverbal.

### 3.6. GIS and Challenging Behavior

Challenging behavior can be prevalent in children with ASD. In one study, challenging behavior was identified in 99% of participants, 67% of whom displayed three types of behavior and 28% who displayed two behaviors [58]. Preschool children with ASD with GIS were found to have substantially more internalizing and externalizing symptoms, significantly different emotional/behavioral problems, and restrictive/repetitive behaviors than those without GIS [48]. The number of GIS has also been positively associated with SIB [50], aggression and attention problems [32,50], somatic complaints [50], and affective problems [44]. A large epidemiological study has also found that pica, the repeated ingestion of non-nutritious nonfood items, is present in 9.7% of children with ASD and is associated with vomiting, diarrhea, and loose stools in children with ASD (*n* = 1244) [29].

The presence of challenging behaviors may reflect the expression of abdominal discomfort in preschool children with ASD [48]. Ferguson et al. [14] found age differences in the association between problem behavior and GIS. They examined the relationships between GIS, problem behaviors, and internalizing symptoms in an older group of children with ASD aged from 6 to 18 (M = 9.19; SD = 2.94), and a younger group aged from two to five (M = 3.03; SD = 1.07). They found that children with aggressive behaviors (11.2%) were more likely to experience nausea. In the younger group, aggressive behavior was a significant predictor of nausea (B = 0.106, SE = 0.052, *p* < 0.05), and aggression may be used to communicate GI discomfort [14]. In the older age group, GIS were predicted by several behaviors. Older children with anxiety were 11% more likely to have constipation and 9% less likely to have stomach aches. Those with more withdrawn behavior (10.9%), were more likely to have stomach aches but 8.7% less likely to experience constipation. Whereas children with somatic complaints were 11.4% more likely to experience nausea and 11.5% more likely to experience stomach aches. Further evidence that verbal ability affects the presence of challenging behavior is available from a study that involved verbal children with ASD (n = 136) who did not have coexisting cognitive deficiency. This study found that twice as many participants with GIS, compared to those without GIS, exceeded the borderline clinical range for affective problems. GIS were associated with abdominal pain, but group scores on total internalizing or externalizing problem scores did not differ [44]. These findings suggest that a verbal participant may effectively communicate their GI discomfort and receive support before the GIS impacts their behavioral control [44].

Studies that have evaluated the effect of interventions provide evidence of a causal relationship between GIS and challenging behavior. Ghalichi et al. [32] conducted a small RCT that investigated the effect of a six-week gluten-free diet on GIS and behavioral indices in children with ASD (*n* = 80). Data was provided through parental reporting, and parents were unblinded to the intervention. This study found that GIS decreased significantly by 23.47%, and behavioral disorders also decreased significantly during the same period (80.03 ± 14.07 vs.75.82 ± 15.37, *p* < 0.05). During the same period, there was an insignificant increase in the control group who took a regular diet (79.92 ± 15.49 vs. 80.92 ± 16.24). Sanctuary et al. [53] also found that participants after taking a combined prebiotic probiotic supplement for five weeks experienced reduction in at least one GIS and irritability (−6.375, 95% CI (−9.717, −3.033), *p* = 0.003), stereotypy (−3.0, 95% CI (−4.843, −1.158), *p* = 0.006), hyperactivity (−6.25, 95% CI (−10.216, −2.284), *p* = 0.007). This small randomized double-blind controlled trial found that the supplement was well-tolerated in children with ASD (*n* = 8) aged 2 to 11 years old.

### 3.7. GIS and Comorbid Psychopathology

GIS correlates with anxiety and somatic complaints [30]. Fulceri et al. [30] investigated the prevalence of GIS and comorbid problems in preschool children (*n* = 230). They investigated four groups: children with ASD with GIS, children with ASD without GIS, TD children with GIS and without GIS. GIS and comorbid problems were documented by parental report using the Child Behavior Check List. The results indicated a higher percentage of GIS in preschoolers with ASD (37.4%) than in those of TD (14.8%). They found that more anxiety, somatic complaints, externalizing, and total behavioral problems were reported in children with ASD with GIS than in those without.

Williams et al. [58] investigated the relationship between anxiety and GIS, sleep problems, and challenging behavior in children with ASD and adolescents (*n* = 109) aged between 6 and 17 years old. Within this sample, 25% had a diagnosed anxiety disorder, and 75% were in the clinical range for anxiety. The recruitment of informant parents was through online forums and data on anxiety symptoms, and GIS were collected using the DSM-Oriented Anxiety Problems scale from the Child Behavior Checklist and the Gastrointestinal Symptom Inventory, respectively. This study had good statistical power, and the results indicated that higher levels of anxiety were associated with higher levels of GIS. They found significant positive correlations between anxiety and GIS. A small positive correlation existed between anxiety and nausea, anxiety and constipation, ((r (109) = 0.19, *p* < 0.05) and (r (109) = 0.20, *p* < 0.05), respectively. Anxiety also significantly correlated with sleep problems, and the latter were the strongest predictor of anxiety. However, no relationship between anxiety and challenging behavior was detected.

Neuhaus et al. [46] conducted secondary analysis on GIS and psychiatric symptoms data obtained from the Simons Simplex Collection. Data was from children and adolescents with ASD (n = 2756), aged between 4 and 18 years old. They found that behaviors associated with GIS differed between children aged 4–10 years and those over 10–18 years. With younger children, GIS accounted for (1% of the variance, F(1, 1671) = 16.42, *p* < 0.001), and externalizing behavior was higher with higher levels of autistic symptoms. In contrast, in older children GIS, 1.4% of variance, (F(1, 898) = 14.64, *p* < 0.001), and levels of externalizing behavior were lower. To conclude, limited variance in psychiatric outcomes was attributed to GIS, but the link was higher in younger children who had more GIS and lower levels of adaptive behavior.

### 3.8. GIS and Sleep Problems

The presence of sleep problems is associated with lower levels of social cognition, daily living skills, and GIS [47,51,59]. Neumeyer et al. [47] found that constipation correlated with sleep disorders in younger children (6.24%; O/E = 1.62; *p* < 0.001), and the number of GIS individuals with ASD was positively associated with parasomnias and reduced sleep duration [50]. Yang et al. [59] investigated the association between GIS and sleep problems over three years with Chinese children with ASD (*n* = 169) and healthy children (*n* = 172), aged 3–12 years. They found GIS in ASD was associated with maternal sleep problems during pregnancy, children being “picky eaters”, and bottle feeding.

McCue et al. [45] used secondary data analysis of data collected through the autism genetic resource exchange AGRE project from children with ASD (*n* = 610) aged 2–18 years. They examined GI dysfunctions as a risk factor for sleep disorders in children with idiopathic ASD. The AGRE database possessed two variables that contained data on GI dysfunctions. The results of the study indicated that the adjusted odds ratio for sleep disorders amongst GI dysfunctions compared with those without GI dysfunctions was 1.74 (95% CI: 1.22–2.48). The odds of experiencing multiple sleep disorder symptoms among those with GI dysfunctions, when adjusted for age, gender, behavioral problems, bed wetting, current and past supplements, and current and past medications for ASD, were 1.75 (95% CI: 1.10–2.79) when compared with those in children with no GI dysfunctions. Children with ASD with GI dysfunction were nearly twice as likely to have multiple sleep disorder symptoms if they had GI dysfunctions. The authors concluded that the early detection and treatment of GI dysfunctions amongst those with ASD may be a solution to reducing the prevalence and severity of sleep problems experienced by those with ASD.

### 3.9. GIS and Sensory Issues

Food selectivity, which involves the intake of restricted types of foods, and atypical eating behaviors are common among children with ASD and adolescents [27]. Poor appetite and constipation were positively related to atypical eating behavior in 16.7% of a large sample of children with ASD and adolescents (*n* = 1112) [60]. However, in this study, the correlations between atypical eating behaviors, and stomach aches, nausea, vomiting, diarrhea, and bowel soiling accidents were insignificant.

Food selectivity has been associated with the presence of at least one GIS, higher levels of SIB, and anxiety problems (*p* < 0.001 Cohen’s *d* = 0.65) [48]. Esposito et al. [27] investigated the factors associated with food selectivity amongst children with ASD (*n* = 50, 84% male) and NT (*n* = 50, 78% male). Children’s eating behaviors and sensory issues were measured using the Brief Autism Mealtime Behavior Inventory, and the Short Sensory Profile [64], respectively. Parental feeding habits were also examined. The results revealed that children with ASD displayed more levels of food selectivity than NT did and that this was secondary to sensory anomalies. Children with ASD had significantly different sensory profiles to the TD group regarding tactile desensitivity, under-responsive/seek sensation, gustatory movement, and visual hypersensitivity. In addition, food refusal was associated with lower BMI, sensory issues, and dysfunctional behavior. They concluded that food selectivity involves GIS, individual hypersensitivity, parental behavior, and environmental factors.

Sensory processing and impairment were also examined by Khalil et al. [40] who investigated the relationship of GIS to the presence of *Clostridium difficile* in the gut of children with ASD (*n* = 59), TD siblings (*n* = 45), and a control group (*n* = 45). The Short Sensory Profile (SSP) was used to evaluate sensory issues, and quantitative real-time PCR was done to analyze *Clostridium difficile* and its toxins A and B. *Clostridium difficile* was detected in children with ASD (25.9%), siblings (40%), and unrelated controls (15.6%), respectively. There was no outstanding difference between the qualitative, quantitative, and toxin production between groups of participants. There was definite sensory impairment in 89.7% of participants, and 10.3% had sensory impairment that made a “probable difference”. The highest levels of impairments were found in the “under-responsiveness/seek sensation” category (91.4%). However, *Clostridium difficile* was not prevalent in the gut of the participants with ASD. Despite most of the strains being toxigenic, there were no GIS recorded in the control groups and no notable association with GI Severity Index in autistic participants.

Leader et al. [41] also investigated sensory issues and the relationship between feeding problems and GIS in children and adolescents (*n* = 120) aged between 3–17 years old. Feeding problems included food selectivity, disruptive mealtime behaviors, food refusal, and limited food intake. They found that sensory issues were a partial predictor of food selectivity, which was common and exhibited by 84.6% of their sample. GIS were predictive of chewing problems, rapid eating, and vomiting. They concluded that feeding problems are associated with sensory issues and that they highly impact the quality of life and the adaptive functioning of autistic participants.

## 4. Discussion

This paper has systematically reviewed the peer-reviewed literature published since 2014 on the frequency and nature of GIS in children with ASD and adolescents and their relationship to regression in ASD, ASD severity language and communication, challenging behavior, comorbid psychopathology, sensory issues, and sleep problems. Table 2 summarizes the findings of this review regarding how key factors are associated with the presentation of GIS in autistic children and adolescents.

The evidence reveals that GIS are more common in children with ASD and adolescents than in children with TD and that GIS are unrelated to age and persistent over time. There is a large variation between the rates of GIS prevalence reported by individual studies, as has been previously noted [6]. This review also reveals contradictory evidence on whether the prevalence of GIS is associated with gender.

Over half of the studies that examined the association between ASD symptoms and GIS concluded that GIS did not have a significant association with ASD severity [36,52,53], but other studies reported that GIS was associated with ASD severity. Studies prior to 2014 have also described contradictory results [4]. Therefore, definite conclusions concerning the relationship between ASD severity and GIS are yet to be drawn.

There is limited but valuable data from Reynolds et al. [51] supporting the existence of an association between GIS and regression in ASD. These findings concur with previous research. Valicenti-McDermott et al. (2008) [17] found that children with ASD who presented with language regression had more GIS than those without language regression. Similarly, Niehus and Lord (2006) [65] found that the medical records of children with ASD and regression indicated significantly more parental reports of bloody stools than of those with ASD and no regression did. However, Reynolds et al. [51] contrasts with other research in which developmental regression was not significantly associated with GIS [18,19].

Evidence concerning the relationship between GIS and language and communication is also somewhat inconclusive. There was no difference in the verbal ability of children with and without GIS [49], and yet constipation was associated with speech problems and feeding problems [47]. This finding concurs with earlier research that children with ASD with GIS showed higher levels of social impairment than those without GIS [20]. This latter study also found that younger, more socially impaired and nonverbal children had increased odds of constipation. However, other previous research found no difference between the verbal ability of children with and without reported pain abdominal pain, or constipation [18], or rates of GI complaints [21].

The presence of challenging behaviors is associated with the presence of GIS, especially those with a reduced verbal ability [14]. Challenging behaviors are common, and they occur in between 40% and 65% of children with ASD who have at least one GIS [14,44,48]. The recent research findings align with previous research that found GIS were not significant predictors of aggression [66], and tantrum behaviors were more common in those with GIS [67] and are predicted by diarrhea [9]. Previous research also found that nausea predicted conduct, avoidant and worry/depressed behavior, and constipation and abdominal pain predicted conduct behavior [9]. SIB was not, however, associated with GIS [67].

Recent research regarding GIS and comorbid psychopathology has added valuable additional knowledge concerning psychiatric outcomes and particularly concerning anxiety. Leader et al. [41] documented high levels of comorbid psychopathology in ASD, and parents reported atypical levels of anxiety, depression, and poor mental health, and over a third (37.7%) of children with psychiatric symptoms experienced at least one GIS. Anxiety and somatic complaints are associated with GIS [30,58]. However, there was limited variance in psychiatric outcomes due to GIS and more association in younger children with more GIS [46]. This recent research concurs and builds upon previous research that GIS are related to anxiety [21,66,68].

Several studies examined GIS and sensory issues and they revealed that sensory issues are common in ASD [27,40,41,48,60]. The studies report that sensory issues are exhibited more frequently by children with ASD and adolescents in comparison to children with TD [27] and that their presence was associated with increased rates of feeding problems and GIS [41,48]. The highest levels of impairments were found in the “under-responsiveness/seek sensation” category [40]. This finding somewhat contrasts with previous work that found GI problems associated with sensory over-responsivity [66].

The research reviewed on GIS and sleep problems concurs with previous work that sleep problems are common and are associated with GIS [9,45,47,51,59,69,70,71]. The evidence suggests that there is a 68% increase in the odds of an autistic child with GI dysfunction, regardless of age and gender, having at least one sleep disorder symptom in comparison to children who do not have GIS [45]. The prevalence of sleep problems and the increased likelihood of sleep problems in children with GIS has implications for the children and their caregivers. Sleep disorders can exacerbate ASD symptomatology [45], poor health outcomes [69], and are associated with externalizing and internalizing behavior problems [72,73]. It is interesting that sleep problems in children with ASD with GIS were reported to be associated with maternal sleep problems during pregnancy [59]. This suggests the possibility that maternal sleep problems may carry over to children after birth. Lyu et al. [74] also found that maternal sleep duration is positively associated with child sleep duration. However, the relationship between ASD and sleep problems is unclear as there is a lack of longitudinal studies on this topic [75].

### 4.1. Future Research

The vast majority of studies deployed cross-sectional designs that did not allow examination of causality. Some evidence of causal directions is provided through the results of research that has evaluated interventions that aim to alter the gut microbiota through a fecal transplant. However, to date, this promising research has utilized small sample sizes. More research is needed to determine the longer-term effects of GIS in the context of ASD [76] and to understand the mechanisms that underlie the relationships between GIS and ASD. For example, the data concerning the contribution of celiac disease and gluten intolerance is currently controversial. There is limited evidence of an association between celiac disease and ASD [77], and the prevalence of celiac disease was not found to be higher in an ASD population in comparison to that in a control group [78]. However, it may be that in the presence of ASD, celiac disease presents in an atypical way [78]. There is also convincing evidence of a positive and significant association between ASD and allergic conditions, especially food allergy [79]. To provide a more nuanced understanding of the mechanisms that underlie relationships with GIS and the directionality of causal relationships in the context of ASD, future research needs to be prospective and involve larger samples, like that of the GEMMA study [13].

Future studies need to provide more explanation concerning gender differences in the presence and severity of GIS, and whether sex hormones interact with the GI microbiota potentially impacting the gut–immune–brain axis [24,80]. The latter could also be differentially impacted by individuals with subtypes of ASD including regression. Future trials assessing interventions would also benefit from placebos and double-blind designs.

Future research is also needed to guide the management of GIS in children with ASD [76]. It has been recommended that future research focuses on how diagnostic screening and the assessment of GIS can be standardized and improved [76]. In addition, it may be helpful for children with GIS and ASD to have consultations with gastroenterologists [81]. Regarding treatments for GIS in ASD, there is controversy that needs resolution through further investigation. Probiotics have been recommended as a complementary therapy [81], but some studies have found little evidence of the efficacy of nutritional supplements and dietary therapies [82]. Therefore, the efficacy of specific strains, their optimal dosage, and the duration of treatments remain to be established [83].

Current research assesses GIS using a large variety of tools. This makes meta-analysis and comparison of their results problematic. Several studies used the gastrointestinal severity index [24,40,54,78] or the Autism Treatment Network 2005 Questionnaire [41,43,44,58]. Some studies used validated questionnaires, but several others measured GIS using questionnaires that were unique to wider studies. In order to improve standardization and accurate assessment, future research needs to develop and use structured and validated tools.

The majority of recent research measures GIS using parental reports. In only a few studies there were interviews with parents conducted by clinicians. Parental reports are unlikely to accurately report less visible GIS including nausea and abdominal pain. Selection bias is also likely, as parents whose children have GIS are more likely to be interested and forthcoming as informants. Therefore, use of multiple data sources in studies, including reporting from children, parents, and clinician examination, are likely to improve the validity of future research. The accuracy of GIS measurements will also improve if future work develops consensus regarding how GIS are defined and if these definitions are clearly communicated to informants. Most current studies do not report how they defined GIS for informants. When definitions were provided, they varied subtly between the studies. For example, constipation was defined as defecation less than two times/week [24]; no defecation for more than three days [27], and two or more hard stools per week [51]. Some research used tools that are likely to improve the accuracy of measurements. For example, food diaries were used [34], as was the Bristol stool chart [34,51,53]. The latter facilitates informants to report stool consistency.

Future work also needs to increase consensus regarding the time intervals during which GIS are evaluated. Many studies do not report the time interval, but others reported that they measured GIS experienced in the preceding three months. However, it has been argued that more timely informative data would be provided if GIS are recorded if they are present in the previous month rather than three months [41].

### 4.2. Review Strengths and Limitations

This review used a systematic methodology with good inter-rater agreement on procedures. Appraisal of the strengths and weaknesses of the studies against the QuADS criteria resulted in one study being rated moderate (11–20), 28 rated moderately high quality (rated 21–30), and one study was rated high quality (rated 31–39). However, because the review only included studies published in English, potentially informative studies may have not been included in this review.

## 5. Conclusions

This review systematically examined the current literature to increase the understanding of the prevalence and nature of GIS and its relationship to co-occurring conditions in children with ASD and adolescents. The review found that recent literature has focused on the association between GIS and developmental regression, language and communication, ASD severity, challenging behavior, comorbid psychopathology, sleep problems, and sensory issues. Further research is needed to develop a more nuanced understanding of causal pathways and how these symptoms can be identified and treated. Research needs to examine larger samples using prospective longitudinal designs and to measure GIS in objective standardized ways.

## Figures and Tables

**Table 1:** Summary of study details.

Study ID	Study Design	Aim(s)	Sample–Size and Age	Measure of GI Symptoms	Results	QuADS Result
Babinska et al. [24]	Case-control study	Investigated the prevalence and types of GIS, frequency of food selectivity, mealtime difficulties.	*n* = 247 (ASD) *n* = 267 (TD) 2–18 years	GI severity index questionnaire [25].	Higher prevalence GIS in autistic girls than boys. High rates food selectivity 69.1% and mealtime difficulties 64.3%. Weak but significant correlation of behavior characteristics with GIS frequency,	21
Bresnahan et al. [26]	Cohort Study	Investigated GIS frequency in children registered in Norwegian Mother & Child study, and when these present during first 3 years of life.	*n* = 195 (ASD) *n* = 4636 (DD) *n* = 4095 (TD) 6–36 months	Study Maternal report questionnaires.	Mothers of children with ASD were significantly more likely to report constipation, food allergy/intolerance in the 6–18 month and diarrhea, constipation and food allergy/intolerance in the 18–36 month periods.	29
Chaidez, et al. [8]	Population-based case-control study	Compared GI problems in children registered in the CHARGE study examining the relationship between GIS and maladaptive behaviors.	*n* = 499 (ASD) *n* = 137 (DD) *n* = 324 (TD) 24–60 months	CHARGE GI questionnaire	Children with more severe ASD had 10% more frequent diarrhea than those with less severe ASD. ASD children more likely to have at least on frequent GIS than TD. Maladaptive behaviors correlate with GIS.	21
Esposito et al. [27]	Case-control study	To clarify key factors associated with food selectivity.	*n* = 41 (ASD) *n* = 48 (TD) 25–98 months	Questionnaire interviews [28]	Parenting style, sensory anomalies, and GIS were associated with food refusal of children. Presence of GIS were associated with hypersensitivity to smell, and moving visual stimuli in ASD children.	26
Ferguson et al. [14]	Cohort study	Examined the relationship between GIS, problem behaviors and internalizing symptoms in children with ASD and adolescents.	*n* = 340 (ASD) 2–18 years	Questionnaires completed at clinic visits by caregivers	Aggression was a predictor of nausea in younger children. Older children with anxiety were 11% more likely to have constipation, 9% less likely to have stomachaches.	23
Fields et al. [29]	Case-control study	Examined the relationship between Pica, GIS, and ASD.	*n* = 1244 (ASD) *n* = 1593 (DD) *n* = 1487 (TD) 2–5 years	Parental information on health history	Pica was associated with vomiting, diarrhea, and loose stools in all groups. Without pica, increased GIS is still evident in ASD.	19
Fulceri et al. [30]	Population-based case-control study	Explored the type and the prevalence of GIS in ASD, TD controls, and investigated their association with behavioral problems.	*n* = 115 (ASD) mean (SD) age = 3.8 (1.1) years and *n* = 115 (TD)	Somatic Scale of Child Behavior Check List 1.5–5 [31]	ASD children with GIS had more anxiety problems, somatic complaints, externalizing, and total problems than those without GIS.	32
Ghalichi, et al. [32]	RCT open design	Investigated the effect of a gluten-free diet on GIS and behavior in ASD children.	*n* = 80 (ASD) 4–16 years	ROME III questionnaire [33]	GI abnormalities in 53.9%. In the gluten-free diet group, the prevalence of GIS decreased significantly to 17.10%. Gluten-free diet resulted in a significant decrease in behavioral disorders (80.03 ± 14.07 vs. 75.82 ± 15.37, *p* < 0.05).	26
Grimaldi et al. [34]	RCT double-blind	Investigated effect of exclusion diet and prebiotic on gut microbiota, metabolism and behavior.	*n* = 30 (ASD) 4–11 years	GIS symptom Diaries Bristol Stool Chart [35]	Significant changes in GIS due to prebiotic, metabolism, and reduction in antisociability scores.	21
Jiang et al. [36]	Case-control study	Investigated the association between GIS and ASD symptom severity and developmental functioning.	*n* = 28 (ASD + GIS) *n* = 28 (ASD) *n* = 28 (DD) *n* = −28 (DD + GIS) 17–37 months	Study clinical information questionnaire.	Although the prevalence of GIS was higher in participants with ASD than those without, this difference was not significant. GIS were unrelated to ASD symptom severity or developmental functioning.	23
Kang et al. [37] Kang et al. [38]	Open-label clinical trial	Investigated the safety and tolerability of microbiota transfer therapy and its effects on microbiota, GIS, and other ASD-related symptoms.	*n* = 18 (ASD) 7–17 years	The GI Symptom Rating Scale [39].	Reduction of 80% and 23% of GIS and ASD severity, respectively, after treatment. Significant improvements in constipation, diarrhea, indigestion, and abdominal pain. Improvements maintained at 8 weeks, and 2-year follow-ups.	22
Khalil et al. [40]	Case-control study	Assessed Clostridium difficile in the stool & its relation to GI comorbidities, ASD severity, and sensory impairment.	*n* = 58 (ASD) *n* = 45 (TD siblings) *n* = 45 (controls) 3–10 years	Short Version of GI Severity Index; 6-GSI Questionnaire [25].	No statistically significant difference between groups in Clostridium difficile, qualitative, quantitative, and toxin production results. Sensory symptoms and GIS are common comorbidities in ASD.	26
Leader et al. [41]	Cohort study	Investigated frequency of feeding problems and their relationship to GIS, challenging behavior, sensory problems and comorbid psychopathology.	*n* = 136 (ASD) 3–17 years	GI Symptom Inventory [42].	Food selectivity was present in 84.6% of the sample. Participants with feeding problems had a higher rate GIS and of challenging behavior and sensory issues.	23
Mannion & Leader [43]	Cohort study	Examined change in comorbid symptoms of ASD over two years and the relationship between symptoms, family medical history, including autoimmune diseases.	*n* = 56 (ASD) 5–19 years	GI Symptom Inventory [42]	GIS persisted in 84.4% of participants and 92.9% had family history of autoimmune disease.	29
Mazefsky, et al. [44]	Cohort study	Explored the association between GIS and emotional behavior concerns in children with ASD without intellectual disability (ID).	*n* = 95 (ASD) 7–19 years	GI Symptom Inventory [42].	Participants with/without GIS did not differ regarding adaptive behavior, or total internalizing or externalizing problem scores but those with GIS had significantly higher levels of affective problems.	27
McCue, et al. [45]	Retrospective cohort study	Investigated whether GI dysfunctions increased the odds of sleep disorders in children with idiopathic ASD.	*n* = 610 (ASD) 2–18 years	Autism Genetic Resource Exchange Data Source, 2013.	Sign more sleep disorder s/s for children with GI problems (64.1%; 150/234) than for those without GI problems (50.8%; 156/307).	24
Neuhaus, et al. [46]	Cohort study	Explored the relationship between GI concerns and psychiatric symptoms in children and adolescents with ASD	*n* = 2756 (ASD) 4–18 years	Parental interviews relating to GI concerns.	Higher levels of psychiatric symptoms were associated with more ASD symptoms, higher verbal IQ, lower family income, and lower adaptive behavior skills. GIS accounted for unique variance in psychiatric outcomes over &above these factors.	26
Neumeyer et al. [47]	Cohort study	Identified associations among co-occurring medical conditions in children with ASD spectrum disorders.	*n* = 2114 (17 months–5 years) *n* = 1221 (6–17 years)	Somatic Scale of Child Behavior Checklist (CBCL/1½–5) [31].	Confirmed association between sleep disorders and anxiety symptoms, in older children; Associations between feeding with sleep disorders (younger children only) and speech disorders; constipation with sleep disorders and speech disorders.	26
Prosperi et al. [48]	Cohort study	Investigated the prevalence and type of GIS and food selectivity (FS) problems and examined the association with ASD severity, cognitive ability, behavioral problems.	*n* = 163 (ASD) (20–71 months)	Somatic Scale of Child Behavior Checklist (CBCL/1½–5) [31].	At least one severe GI symptom or FS in 40.5% of participants. Levels of behavioral problems were significantly different for participants with/without GIS and FS. No significant difference in the performance of IQ and autistic severity.	25
Prosperi et al. [49]	Observational case-control study	Investigated the presence and type of associated verbal and motor behaviors determined their correlation with GIS.	*n* = 85 (ASD) (2.18–6.11 years)	GI Severity Index [25].	GIS group had 35% higher scores in behaviors than the non-GI group.	22
Restrepo et al. [50]	Cohort study	Examined the association of GIS with gender, developmental and behavioral measures.	*n* = 255 (ASD) *n* = 129 (TD) (2–3.5 years)	GI History (CHARGE GH) Questionnaire. [8]	Somatic complaints increased with number of GIS. Children with ASD and co-occurring GIS experienced more behavioral problems than individuals with ASD without GI concerns.	25
Reynolds et al. [51]	Case-control study	Evaluated associations between GIS and neurodevelopmental phenotypes.	*n* = 672 (ASD) *n* = 938 (DDs) *n* = 851 (TD) (2–5 years)	Study GI Questionnaire	Children with ASD with regression had increased odds of several GIS than ASD children without regression.	25
Rose et al. [52]	Cohort study	Investigated biological signatures to immune dysfunction and microbiota composition in ASD children with GIS.	*n* = 103 (ASD) (3–12 years)	CHARGE GI History [8].	The ASD GI group produced increased mucosa-relevant cytokines compared to ASD no GI group.	25
Sanctuary et al. [53]	RCT double-blind	Assessed tolerability of combined probiotic-BCP supplement.	*n* = 8 (ASD) (2–11 years)	CHARGE GI History (Chaidex et al. 2014)	Five weeks of treatment was tolerated well. Children on both treatments saw a reduction in the frequency of certain GIS.	30
Shaaban et al. [54]	Prospective, open-label study	Evaluated the efficacy and tolerability of probiotic supplement.	*n* = 30 (ASD) (5–9 years)	GI Severity Index questionnaire [25].	Reduced GIS after 3 months of therapy, which was well-tolerated.	23
Thuslai et al., [55]	Case-control study	Examined gastrointestinal symptoms in pediatric outpatients.	*n* = 135 (ASD) *n* = 146 (with and without GIS).	GI Severity Index questionnaire [25].	The Gastrointestinal Severity Index was more effective in screening for gastrointestinal disorders in comparison.	21
Tomova et al. [56]	Pilot study	Examined changes in fecal microbiota and determined its role in the development of GI disorders.	*n* = 10 (ASD) *n* = 9 (TD siblings) *n* = 10 controls 2–9 years (ASD) 2–11 years (controls).	Study Parental Questionnaire.	The participants demonstrated strong positive correlation of ASD severity with the severity of GI dysfunction.	21
Vargason et al. [57]	Retrospective cohort study	Examined the association between early antibiotic use and the occurrence of later GIS.	*n* = 3253 (ASD) *n* = 278,370 (Controls).	Analyzed claims from US health insurer for antibiotic use and GI diagnosis.	More antibiotic prescriptions early in life were associated with increased rate of later GI diagnosis (adjusted hazard ratio 1.48; 95% confidence interval 1.34, 1.63) for children with and without ASD	30
Williams, et al. [58]	Cohort study	Examined the relationship between anxiety and GIS, sleep problems and challenging behavior.	*n* = 109 (ASD) 6–17 years	GI Symptom Inventory [42].	Demographic factors, GIS, sleep problems, and challenging behavior accounted for 34% of the variance in anxiety. Sleep, severity of self-injurious behavior, age, and ID diagnosis are significant predictors.	26
Yang et al. [59]	Case-control study	Examined associations between GI and sleep problems, severity of ASD and behavioral symptoms.	*n* = 169 (ASD) *n* = 170 (TD) 3–12 years	Study Clinical Information Questionnaire	ASD + GIS associated with more severe ASD core symptoms than those without GIS. GIS were associated with maternal sleep problems in pregnancy, breast/formula feeding 0–6 months, and picky eating.	23
Zickgraf et al. [60]	Cohort study	Examined psychological, health, and demographic correlates of atypical eating.	*n* = 1112 (ASD) 1–17 years	Pediatric Behavior Scale [61].	Found atypical eating behaviors in 70.5% of participants. These positively related to age (most common at ages 1–3), increasing ASD severity, poor appetite, and constipation.	28

Abbreviations: ASD = autism spectrum disorder, BCP = bovine colostrum product, DD = developmental disability, GI = gastrointestinal, GIS = gastrointestinal symptoms, ID = intellectual disability, IQ = intelligent quotient, RCT = randomized control trial, TD = typically developing.

**Table 2:** Key factors and their association to GIS.

Relationship	Factor	
No Association	Age [8,14,24]	
Contradictory Evidence	Positive Association [47]	Language and Communication Ability
	No Association [49]	
	Positive Association [36,40,44,50]	ASD Severity
	No Association [37,38,52,56,59,60,62]	
	Positive Association [24,44]	Gender
	No Association [48,49,50]	
Positive Association	Presence of ASD [8,24,26].	
	Regression in ASD [51].	
	Comorbid Anxiety [30,58].	
	Sensory Issues [27].	
	Sleep Problems [45,47,51,59].	
	Challenging Behaviors [14,29,32,44,48,50].	
